# Scanning Electron Microscope Image Analysis of Bonding Surfaces following Removal of Composite Resin Restoration Using Er: YAG Laser: *In Vitro* Study

**DOI:** 10.1155/2021/2396392

**Published:** 2021-11-27

**Authors:** Dlsoz Omer Babarasul, Bestoon Mohammed Faraj, Fadil Abdullah Kareem

**Affiliations:** ^1^Department of Conservative Dentistry, College of Dentistry, University of Sulaimani, Madam Mitterrand St., Sulaimani, Iraq; ^2^Department of Pedodontics, Orthodontics and Preventive Dentistry, College of Dentistry, University of Sulaimani, Madam Mitterrand St., Sulaimani, Iraq

## Abstract

It is impossible to remove tooth-colored restorations by mechanical means without unnecessary damage to the adjacent sound tooth structure. This study is aimed at investigating erbium-doped yttrium aluminum garnet (Er: YAG) laser (Hoya ConBio, VersaWave, CA, USA) in removing composite resin restorations and assessing the change in morphology of bonding surfaces using a scanning electron microscope (EDX, CAMSCANNER, 3200LV, UK). The investigators collected thirty extracted sound human premolar teeth for this investigation, and the conventional design class V cavity was prepared on the buccal surface of each specimen. The specimens were allocated randomly into three groups, according to the procedure used for the ablation of the composite restoration: group A (high-speed diamond fissure bur), group B, and group C (Er: YAG laser) using a different pulse repetition rate of 20 Hz (group B) and 25 Hz (group C). The AutoCAD software program (Autodesk, Inc., 2016) was used to calculate the surface area and the resulting dimensional change of the cavities after restoration removal. The cavities were filled with composite resin and randomly assigned into two groups conforming to the methods applied to eliminate the restoration; diamond turbine fissure bur and laser. In each group, two specimens were selected randomly for scanning electron microscope analysis of bonding surfaces. The least meantime for the composite resin removal was observed in the high-speed diamond bur, significantly less than both Er-YAG laser groups (*p* < 0.001). However, at a higher pulse repetition rate, time-consuming decreased. The results showed that laser is more conservative in removing composite resin restoration as the change was most remarkable in group A (0.800 mm), then group C (0.466 mm), and the slightest change is in group B (0.372 mm) (*p* = 0.014). The dentin surface of group A showed a smooth surface with no opened dentinal tubule and intact smear layer. In groups B and C, dentin surfaces were irregular, scaly, or flaky, and dentinal tubules were opened without a smear layer. Therefore, Er: YAG laser is effective for composite resin removal considering the parameters chosen in this study with fewer changes in cavity surface area and better microretentive features.

## 1. Introduction

The concept of minimally invasive dental practice will provide promising approaches for using composite resin restorations, which are difficult to distinguish from the surrounding tooth substance and adhere to the enamel and dentine firmly, making them hard to remove without enamel and dentine destruction. Thus, clinicians commonly remove excessive amounts of sound tooth substances to guarantee the complete removal of composite material [1]. Therefore, a technology that can remove composite selectively and rapidly from tooth surfaces while minimizing the inadvertent removal of sound tooth structure is considered a significant improvement over current methods. However, the clinician also has to adopt an equally conservative approach when treating failed restorations. The quality of the composite resin restoration is determined by the outline form of cavity preparation and the dentist's technique and understanding of the used materials [2, 3].

Despite advances in restorative material technology, dental practitioner still devotes a significant part of their clinical time to repair or replace old restorations. The recent signs of progress in dentistry that have been made enable more proficient, safer work, and predictable management outcomes [4]. Laser is considered one of the brand-new technologies becoming more popular in dental practice and supports conventional treatment forms while replacing traditional treatment modalities. The erbium-doped yttrium aluminum garnet (Er: YAG) laser possesses many favorable results over a conventional cavity preparation by burs [2, 5]. Rough surfaces are irradiated with a laser, resulting in clean and smooth surfaces with opened dentinal tubules and no smear layer [4]. This procedure is essential and approves an exact and entire removal of restorative material penetrating dentin tubules, which is impossible to achieve with a conventional bur [6, 7].

Dental composites are usually color-matched to the tooth for esthetic reasons and are hard to remove by mechanical means without exerting excessive harm to surrounding enamel and dentin. Ideally, lasers are suited for selective ablation to induce minimal sound dental tissue loss when replacing failed restorations and sealants, removing composite adhesives. Different kinds of lasers have been employed in dental practice. However, nowadays, the Er: YAG laser shows superior performance. The use of Er: YAG laser for ablation of composite and cement restorations has been investigated due to its potential to remove selectively dental composite and carious tissue, minimizing inadvertent removal of sound tooth substance and without the creation of the smear layer [8, 9].

The scanning electron microscope (SEM) is broadly used in the in vitro evaluation of materials, including microstructure morphology of bonding surfaces and nanomaterial analysis. It can be considered as a reliable precision investigative tool applied for high-resolution surface topography examination. It has the feature of the considerable depth of field, high resolution, instinctive imaging, strong stereo perception, and wide magnification range, and the specimen to be examined can be rotated and inclined in a three-dimensional view [10, 11].

In the present study, the efficacy of Er: YAG laser for the removal of composite restorations using different pulse repetition rates has been investigated in terms of the time required for restoration removal and conservation of tooth structure. Also, the morphological aspect of the cavity was assessed via a scanning electron microscope.

## 2. Methodology

### 2.1. Study Design and Sample Selection

**Figure 1:**
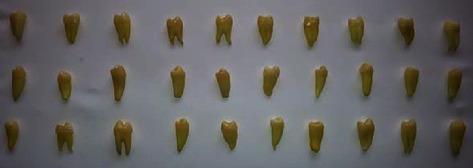
The extracted teeth selected for this study.

The Ethical Committee confirmed the ethical approval for this study at the University of Sulaimani (Ethical No. 68/21). This experiment has delivered the checklist for reporting *in vitro* study (CRIS) guidelines [12]. The investigators selected 30 extracted sound human premolar teeth free from caries, restoration, and crack. Teeth were extracted for orthodontic purposes. A patient consent form and research permissions were obtained to use their extracted teeth in the present research. Specimens were stored in 10% formalin for about two weeks to provide disinfection. A hand scaler was used to remove tissue fragments and calcified debris, then washed and cleaned with tap water. Ultimately, they were stored in distilled water at room temperature until the investigation (Figure 1).

### 2.2. Specimen Preparation

A 30 cylindrical container of approximately (15 mm) was prepared from a disposable plastic syringe (20 ml) which was cut horizontally by using a fine diamond disk mounted on contra angled handpiece, and the base of the container was sealed by a wax sheet. Each specimen's root was covered by a layer of wax and vertically positioned in the center of the container and embedded inside a cold-cure clear acrylic resin (Vertex Castavaria, Netherland) to the level below the cementoenamel junction by using a dental surveyor. The dental analyzer adjusted parallel to the vertical axes of the tooth.

An experienced operator performed all restorative procedures. A standard class V cavity design was drawn on the buccal surface of the clinical crown (2 mm height, 3 mm width), with a (0.5) mechanical pencil using a matrix band with a precut hole of (2 × 3 mm) that was fixed on the tooth with a retainer. These dimensions were calculated with an electronic digital vernier (Mitutoyo Corp., Japan) nearest (0.01 mm.). Cavities were prepared by drilling (1.5 mm depth) with a butt-joint at an external line angle using a high-speed turbine after maintaining the bur at a right angle to the buccal surface of the teeth using the horizontal arm of a surveyor. Cavities were prepared under cooling water, and each bur was replaced after every five cavities. The rotational speed and applied source were standardized for all specimens.

### 2.3. Photographic Image Analysis of the Surface Area of Prepared Cavities

The color photograph of the prepared cavities was taken using a digital camera (Canon, 12.1 MEGA PIXE, SX510 HS, China), which was adapted to the lens of the stereomicroscope with a power of 40× magnification. The AutoCAD software program (Autodesk, Inc., 2016) was used to calculate the surface area of the prepared cavities at a constant light that resembled the transmitted light of the stereomicroscope with continuous electric power. The distance of the sample to the lens of the microscope was fixed at 40 mm.

### 2.4. Restorative Procedures of Prepared Cavities

All cavities were acid-etched using phosphoric acid (Top Dent Etch gel 38%, DAB Dental, Sweden) using all etch techniques for 15 seconds and thoroughly washed by water spray for 10 seconds blot excess water via cotton pell. Then, two conclusive layers of single-bottle adhesive (Clearfil SE bond, Kuraray Dental, New York, USA) were applied, gently air sprayed for 5 seconds, and light-cured for 20 seconds through the use of a visible-light curing device (400-1000 mW/cm^2^) continuous mode. Handling of all materials was carried out based on the manufacturer's instructions. Then, all the prepared cavities were filled in one layer with a shade A1 nanohybrid composite resin (Tetric Evoceram, Ivoclar Vivadent, New York, USA) using a nonstick titanium-coated applicator and light-cured with LED light cure device (LED Flash max P3 Hexagon, Denmark) with an output power of 400-1000 mW/cm^2^ for 20 sec. All the restored teeth were stored for one week in 37°C distilled water inside an incubator. After that, the samples were exposed to thermocycling and subjected to 500 cycles in between 5°C and 55°C, with 30 sec dwell time [13].

### 2.5. Specimen Grouping

The specimens were allocated randomly into three groups, according to the procedure used for the ablation of the composite restoration.

#### 2.5.1. Group A: (High-Speed Diamond Fissure Bur)

The composite restorations were removed using a high-speed straight flat end diamond fissure bur (6847KR, 018, Komet, Besigheim, Germany) under a constant water spray coolant. The burs were discarded after each use. The speed of bur rotation and amount of water spray was standard for all specimens. A visual examination was used to determine the completion of restoration removal in all groups [14].

#### 2.5.2. Group B and Group C (Er: YAG Laser)

**Figure 2:**
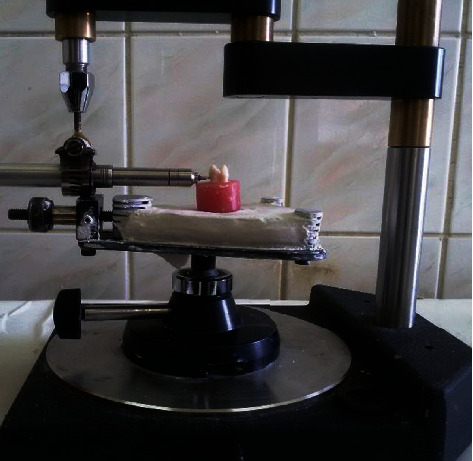
The laser beam kept perpendicular to the target by adapting the handpiece to the horizontal arm of a surveyor.

The employed laser equipment was the Er: YAG (Hoya ConBio, VersaWave, CA, USA), at a wavelength of 2.94 *μ*m, power of 4 W, with a focused beam of 180 mJ energy, with a tip size: 0.8 mm sapphire tip, using a different pulse repetition rate of 20 Hz (group B) and 25 Hz (group C). The irradiation in both subgroups was used under a continuous air-water cooling (15 ml/min), pulse width > 300 *μ*s at a distance of 2 mm from the target surface. The laser beam was kept perpendicular to the target surface by adapting the handpiece to the horizontal arm of a dental surveyor in the way that alternately moved in both right-to-left directions, thus, allowing the laser beam to act on the whole composite restoration. The spot size was measured to be about 0.5 mm^2^ which corresponds to a spot diameter of about 0.8 mm. The unit of measure for energy density was calculated as mJ/mm^2^ (Figure 2).

#### 2.5.3. Evaluation of Time Consumption and Change in Cavity Surface Area (CCSA) following Restoration Removal

The required time for each restoration removal of all studied groups was measured and recorded by stopwatch (Shanghai Diamond Stopwatch, Shanghai, China). After removing restorations, the color photograph of the cavities was taken using a digital camera adapted to the lens of the stereomicroscope, which was adjusted at 40× magnification. The change in surface area was determined according to the following equation: CCSA = cavity surface area before restoration − cavity surface area after restoration removal.

#### 2.5.4. Scanning Electron Microscopic (SEM) Analyses

**Figure 3:**
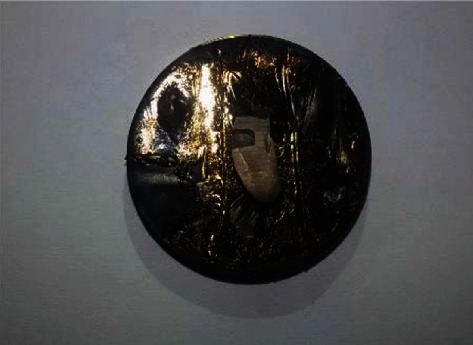
Specimen coated with gold.

SEM analysis was used to evaluate the morphological aspect of the cavities after restoration removal under different experimental conditions. Two samples were selected randomly for scanning electron microscope examination (EDX, CAMSCANNER, 3200LV, UK). First, the tooth was sectioned buccolingual at the center by a diamond disk attached to a straight handpiece under water coolant to permit inspection of the cavity walls (Figure 3). Then, the samples were fixed by mounting on the aluminum stub and coated with gold atoms by using a gold sputter machine. Next, the surfaces were examined qualitatively with a scanning electron microscope operating at 25 kV. A standardized series of photomicrographs were taken with the same magnification (4,000×) in all specimens. Two operators reached a consensus to select the representative illustrations of each group [15].

#### 2.5.5. Statistical Analysis

Data were analyzed using IBM SPSS Statistics for Windows, version 24.0 (Armonk, NY: IBM) software. A *p* value of ≤ 0.05 was considered statistically significant. The sample size was calculated using the Sealed Envelope software for a power of 80%. The normal distribution of the sample was examined using the Shapiro-Wilk test. Paired *t*-test was used to compare the surface area readings before composite placement and after the composite filling removal. Analysis of variance (ANOVA) and *t*-test were used to compare the means of the three study groups. The Cohen's D formula was used to calculate the effect size.

## 3. Results

The least meantime (63.1 seconds) for composite restoration removal was in group A (high-speed diamond fissure bur), which was significantly less than the meantime of group B (Er: YAG laser, 20 Hz), which was 121 seconds, and meantime of group C (Er: YAG laser, 25 Hz) which was 91 seconds as in Table 1. The difference between group B and group C was also significant (*p* < 0.001), as shown in Table 2. Significant differences were observed between the three study groups (*p* = 0.014). The change is most remarkable in group A (0.800 mm), then group C (0.466 mm), and the slightest change is in group B (0.372 mm). The differences between groups A, B, and C were significant (*p* = 0.005 and *p* = 0.026, respectively), while no significant differences were detected between group B and C (*p* = 0.513) as in Tables 1 and 2.  Table 3 shows a considerable increase in the surface area after removing the composite restoration in each of the study groups.

**Figure 4:**
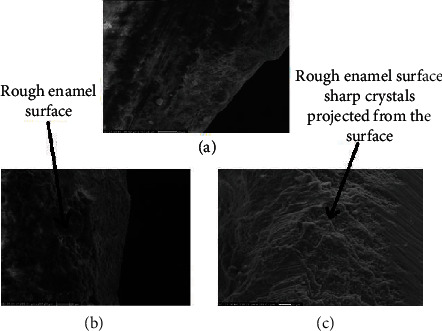
Representative SEM photomicrograph of enamel of the cavity of all groups. (a) Image of group A at magnification (4,000×). (b) Image of group B at magnification (4,000×). (c) Image of group C at magnification (4,000×).

**Figure 5:**
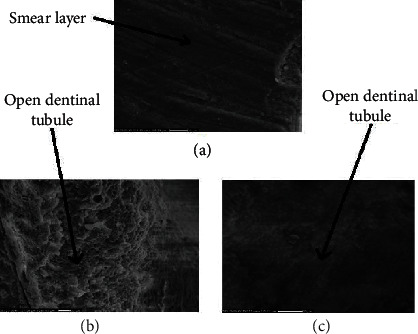
Representative SEM photomicrograph of dentin of the cavity of all groups. (a) Image of group A at magnification (4,000×). (b) Image of group B at magnification (4,000×). (c) Image of group C at magnification (4,000×).

According to representative SEM images findings of the cavity walls at high magnification (4,000×), the enamel in group A (bur group) shows a smooth surface. In contrast, the enamel in groups B and C (laser groups) shows a rough surface and contains microretentive cavities, and in all studied groups, there is no sign of carbonization (black spot). The dentin surface of group A showed a smooth surface with no opened dentinal tubule and intact smear layer. In groups B and C, dentin surfaces were irregular, scaly, or flaky, and dentinal tubules were opened without a smear layer (Figure 4). Representative SEM photomicrograph of the dentin at high magnification (4,000×), the image of group A showed a smooth surface in which the surfaces with no opened dentinal tubule. Pictures of groups B and C show dentin surfaces irregular and surface with the opened dentinal tubules without smear layer, and protrusion of peritubular dentin was revealed (Figure 5).

## 4. Discussion

The present study casts a new light on the efficiency of Er: YAG laser in the removal of composite resin restoration in terms of time consumption, preservation of sound tooth structure, and morphological analysis of final aspects of the cavities after restoration removal. Different parameters such as power output and pulse frequency had to be mentioned to improve laser efficiency. Previous studies had displayed the impact of pulse frequency on ablation rate in cavity preparations. More substance is ultimately removed during the same time interval by increasing frequency, resulting in decreased operation time [16].

The present study showed that the least mean time needed to remove the composite filling was in the bur group, which was significantly less than the meantime of both laser groups. A similar conclusion was reached by other researchers when they found that laser ablation caused longer cavity preparation time than a bur [15, 17]. Regarding the time needed for filling removal, the statistical analysis shows that the difference between group B and group C was significant. Furthermore, in a higher pulse repetition rate, the meantime needed to remove composite restoration was less than the meantime of lower pulse repetition rate. The results show that higher pulse repetition rates required shorter times to remove the composite restorations, although higher pulse repetition rates result in a more substantial temperature increase. These might be interpreted by the fact that the more the pulse repetition rate (i.e., the number of pulses emitted per second), the cooling time will be less in between laser pulses, causing more heat generation.

The present study confirmed the findings of the selective removal of tooth-colored restoration and suggested that the bur sacrificed more tooth tissue, and the laser was more conservative and caused less enlargement of the cavities during removal of the restoration. These results go beyond previous reports, showing that laser is more conservative than bur. The difficulty of ablative process control demonstrated when the greater pulse frequency was used also caused ablation of surrounding sound tissue, mainly deep walls [18, 19]. When Er: YAG laser was used to ablating composite resin restoration surrounded by enamel, specific selectivity for the ablation of composites was displayed, as ablation of enamel is slower than that of composites. However, this selectivity is compromised in dentin because of the higher water content of the dentine. Therefore, the ablation rate of dentin is higher than some composite brands and consistent with what has been found in the previous study [20]. The difficulty of ablative process control observed when the greater pulse frequency was used also caused ablation of surrounding healthy tissue, mainly deep walls.

In this study, the result shows that there was a significant increase in the surface area after removal of the composite restoration in all groups, which agrees with the (Satterthwaite et al., 2009) as they found that all operative interventions carry the risk of additional damage to remaining natural tissues, this will lead to unwarranted removal of healthy tooth substances [21]. So, replacing failed restorations leads to a more extensive cavity outline and weakens the remaining dental substance [22].

Although the present findings show that the laser is more conservative than bur, the cavity size still increased during the restoration's removal. Unskilled ablation causes the enlargement of the cavity preparation size, so demanding care and ability in the use of the laser. According to SEM finding at low magnification bur group show box-shaped configuration while laser groups evidence irregular surface pattern, in low magnification complete removal of composite seen in bur group while the laser group shows incomplete composite removal these findings come under the conclusion of the previous study performed by Correa_Afonso et al. (2010) [15]. As reported by SEM findings, the micrograph of the enamel of the cavity of the bur group shows a relatively smooth enamel surface with mainly exposed, closed prisms, and smooth surfaces consistent with the results of the previous study recorded by Rodríguez-Vilchis et al. (2011) [23]. In laser groups, irregularities are observed, and sharp crystals project from the surface due to ablation. In addition, there was the absence of carbonization and fusion of the enamel structures, which other investigators have also observed from cavity preparation studies in hard dental tissues [23]. Micromorphology of the Er: YAG laser-treated enamel portrays a retentive pattern that resembles acid-etched enamel tissue while anatomical characters of enamel rods are preserved. As a result, irradiated enamel by Er: YAG laser at sufficient output energy provided better marginal integrity of composite resin restorations when compared to a mechanical cutting instrument.

Conforming to SEM findings concerning the bur group, the micrograph of the dentin displayed a smooth surface with no opened dentinal tubule and the presence of a smear layer, in agreement with the result of the previous studies using different energy settings of Er: YAG laser and bur [24, 25].

Despite the feature of a water-mediated photomechanical interaction during hard dental tissue ablation with Er: YAG laser, the mechanism of composite resin ablation includes explosive vaporization followed by hydrodynamic ejection. During ablation of composite resin, rapid melting induces large expansion forces due to the change in volume of the material upon melting. Additionally, surface protrusions are formed, resulting from the counteracting forces combined with the composite resin structure, which are accelerated away from the surface as droplets.

The morphology of dentin after application of Er: YAG laser for cavity preparation possesses an irregular surface with no cracking or fissuring, lack of smear layer, and open tubules, which are considered as a prerequisite for adhesive fixation. These features were responsible for rendering this surface suitable for bonding the resin. This morphological property is a consequence of the high-water absorption wavelength of Er: YAG radiation as a critical determinant for the type of interaction the laser energy is going to have with the target tissue. Bonding resin composites to the dental hard tissues was considered one of the most important contributions to restorative dentistry. Er: YAG laser on dental hard tissues has been regarded as effective and efficient without causing thermal destruction to the adjacent tissue and the dental pulp.

The approach utilized suffers from the limitation that the sample size was small in this study, and the process of restorations removal was limited to class V cavity design. Different cavity designs will compensate for case difficulties and may exhibit further findings regarding more complex tooth-colored restoration concentrating on the time consumption and selective removal of composite restorations.

## 5. Conclusion

The applied treatment protocol that resulted in a minimum change in cavity surface area and microretentive feature in both enamel and dentine responsible for strong adhesive bonding could be generalized to clinical practice. In addition, it may enhance a more predictable treatment outcome.

## Figures and Tables

**Table 1:** Descriptive statistics of time needed to remove the composite resin restoration and the cavity area change in all studied groups.

Variables	Groups	*N*	Mean ± SD	SE	Minimum (sec.)	Maximum (sec.)
Time (second)	A	10	63.100 ± 6.385	2.019	55.0	70.0
	B	10	121.000 ± 9.944	3.145	110.0	140.0
	C	10	91.00 ± 13.904	4.397	70.0	110.0
	Total	30	91.700 ± 26.107	4.767	55.0	140.0

Cavity surface area change (mm)	A	10	0.800 ± 0.331	0.105	0.281	1.189
	B	10	0.372 ± 0.284	0.090	0.010	0.946
	C	10	0.466 ± 0.332	0.105	0.047	0.948
	Total	30	0.546 ± 0.358	0.065	0.010	1.189

**Table 2:** ANOVA and post hock test results for time needed to remove the composite resin restoration and the cavity area change for all studies groups.

Variables	Groups	*p* value	Post hoc	*p* value
Time (second)	A	<0.001	AXB	<0.001
	B		AXC	<0.001
	C		BXC	<0.001

Cavity surface area change (mm)	A	0.014	AXB	0.005
	B		AXC	0.026
	C		BXC	0.513

**Table 3:** Mean, SD, and *t*-test of surface area before and after removal of composite resin restoration of three studied groups.

Groups	*N*	Surface area	Mean ± SD	*p* value
A	10	Before	5.916 ± 0.132	<0.001
	10	After	6.516 ± 0.316	

B	10	Before	5.962 ± 0.188	0.003
	10	After	6.334 ± 0.307	

C	10	Before	5.973 ± 0.192	0.002
	10	After	6.438 ± 0.287	

## Data Availability

Data can be available upon request.

## Abbreviations

CAD-CAM: Computer-aided design-computer aided manufacture
CCSA: Change in cavity surface area
CRIS: Checklist for reporting *in vitro* study
LED: Light-emitting diode
SEM: Scanning electron microscope.

## References

[1] Blum I. R. (2019). Restoration repair as a contemporary approach to tooth Preservation. *Primary Dental Journal*.

[2] Luke A. M., Mathew S., Altawash M. M., Madan B. M. (2019). Lasers: a review with their applications in oral medicine. *Journal of Lasers in Medical Sciences*.

[3] Zakrzewski W., Dobrzynski M., Kuropka P. (2020). Removal of composite restoration from the root surface in the cervical region using Er: YAG laser and drill-in vitro study. *Materials*.

[4] Grzech-Leśniak K., Nowicka J., Pajączkowska M. (2019). Effects of Nd:YAG laser irradiation on the growth of Candida albicans and Streptococcus mutans: in vitro study. *Lasers in Medical Science*.

[5] Perveen A., Molardi C., Fornaini C. (2018). Applications of laser welding in dentistry: a state-of-the-art review. *Micromachines*.

[6] Matys J., Hadzik J., Dominiak M. (2017). Schneiderian Membrane perforation rate and increase in bone temperature during maxillary sinus floor elevation by means of Er. *Implant Dent*.

[7] Yazici A. R., Baseren M., Gorucu J. (2010). Clinical comparison of bur- and laser-prepared minimally invasive occlusal resin composite restorations: two-year follow-up. *Operative Dentistry*.

[8] Korkut E., Torlak E., Gezgin O., Özer H., Sener Y. (2018). Antibacterial and smear layer removal efficacy of Er:YAG laser irradiation by photon-induced photoacoustic streaming in primary molar root canals: a preliminary study. *Photomedicine and Laser Surgery*.

[9] Kornblit R., Trapani D., Bossù M., Muller-Bolla M., Rocca J. P., Polimeni A. (2008). The use of erbium: YAG laser for caries removal in paediatric patients following minimally invasive dentistry concepts. *European Journal of Paediatric Dentistry*.

[10] Meng C., Zhang H. (2020). Scanning electron microscope in metallic materials. *Acta Microscpica*.

[11] Sun C., Lux S., Müller E., Meffert M., Gerthsen D. (2020). Versatile application of a modern scanning electron microscope for materials characterization. *Journal of Materials Science*.

[12] Mirseifinejad R., Tabrizizade M., Davari A., Mehravar F. (2017). Efficacy of different root canal irrigants on smear layer removal after post space preparation: a scanning electron microscopy evaluation. *Iranian Endodontic Journal*.

[13] Krithikadatta J., Datta M., Gopikrishna V. (2014). CRIS guidelines (checklist for reporting in-vitro studies): a concept note on the need for standardized guidelines for improving quality and transparency in reporting *in-vitro* studies in experimental dental research. *Journal of Conservative Dentistry*.

[14] Narayana V., Ashwathanarayana S., Nadig G., Rudraswamy S., Doggalli N., Vijai S. (2014). Asstessment of microleakage in class II cavities having gingival wall in cementum using three different posterior composites. *Journal of International Oral Health*.

[15] Correa-Afonso A. M., Palma-Dibb R. G., Pécora J. D. (2010). Composite filling removal with erbium:yttrium–aluminum–garnet laser: morphological analyses. *Lasers in Medical Science*.

[16] Correa-Afonso A. M., Pécora J. D., Palma-Dibb R. G. (2008). Influence of pulse repetition rate on temperature rise and working time during composite Filling removal with the Er:YAG laser. *Photomedicine and Laser Surgery*.

[17] Rizcalla N., Bader C., Bortolotto T., Krejci I. (2012). Improving the efficiency of an Er: YAG laser on enamel and dentin. *Quintessence International*.

[18] Hjertton P. M., Bågesund M. (2013). Er: YAG laser or high-speed bur for cavity preparation in adolescents. *Acta Odontologica Scandinavica*.

[19] Eberhard J., Bode K., Hedderich J., Jepsen S. (2008). Cavity size difference after caries removal by a fluorescence-controlled Er:YAG laser and by conventional bur treatment. *Clinical Oral Investigations*.

[20] Nevesa A., Coutinhob E., Cardosoc M. V., Lambrechtsd P., Meerbeeke B. V. (2011). Current concepts and techniques for caries excavation and adhesion to residual dentin. *The Journal of Adhesive Dentistry*.

[21] Bader C., Krejci I. (2006). Indications and limitations of Er: YAG laser applications in dentistry. *American Journal of Dentistry*.

[22] Satterthwaite J., Morrow L., Brunton P. (2009). *Principles of Operative Dentistry*.

[23] Rodríguez-Vilchis L. E., Contreras-Bulnes R., Olea-Mejìa O. F., Sánchez-Flores I., Centeno-Pedraza C. (2011). Morphological and structural changes on human dental enamel after Er:YAG laser irradiation: AFM, SEM, and EDS evaluation. *Photomedicine and Laser Surgery*.

[24] Pavithra R., Sugavanesh P., Lalithambigai G., Arunkulandaivelu T., Madan Kumar P. D. (2016). Comparison of microhardness and micromorphology of enamel following a fissurotomy procedure using three different laser systems: an in vitro study. *Journal of Dental Lasers*.

[25] Assaf C., Mouchantaf E., Nasser A. (2015). Er: YAG laser and recurrent caries– a review. *Dental News*.

